# Correction: All^2^: A tool for selecting mosaic mutations from comprehensive multi-cell comparisons

**DOI:** 10.1371/journal.pcbi.1010703

**Published:** 2022-11-15

**Authors:** Vivekananda Sarangi, Yeongjun Jang, Milovan Suvakov, Taejeong Bae, Liana Fasching, Shobana Sekar, Livia Tomasini, Jessica Mariani, Flora M. Vaccarino, Alexej Abyzov

There is an error in reference 11. The correct reference is:

Tu K, Lu K, Zhang Q, Huang W, Xie D. Accurate single-cell genotyping utilizing information from the local genome territory. Nucleic Acids Res. 2021 Jun 4;49(10):e57. doi: 10.1093/nar/gkab106. PMID: 33619552.
