# Supplementary figures and images for: All2: A tool for selecting mosaic mutations from comprehensive multi-cell comparisons
Source: PLoS Comput Biol. 2022 Apr 20;18(4):e1009487. doi: 10.1371/journal.pcbi.1009487 (PMC9060341; doi:10.1371/journal.pcbi.1009487)

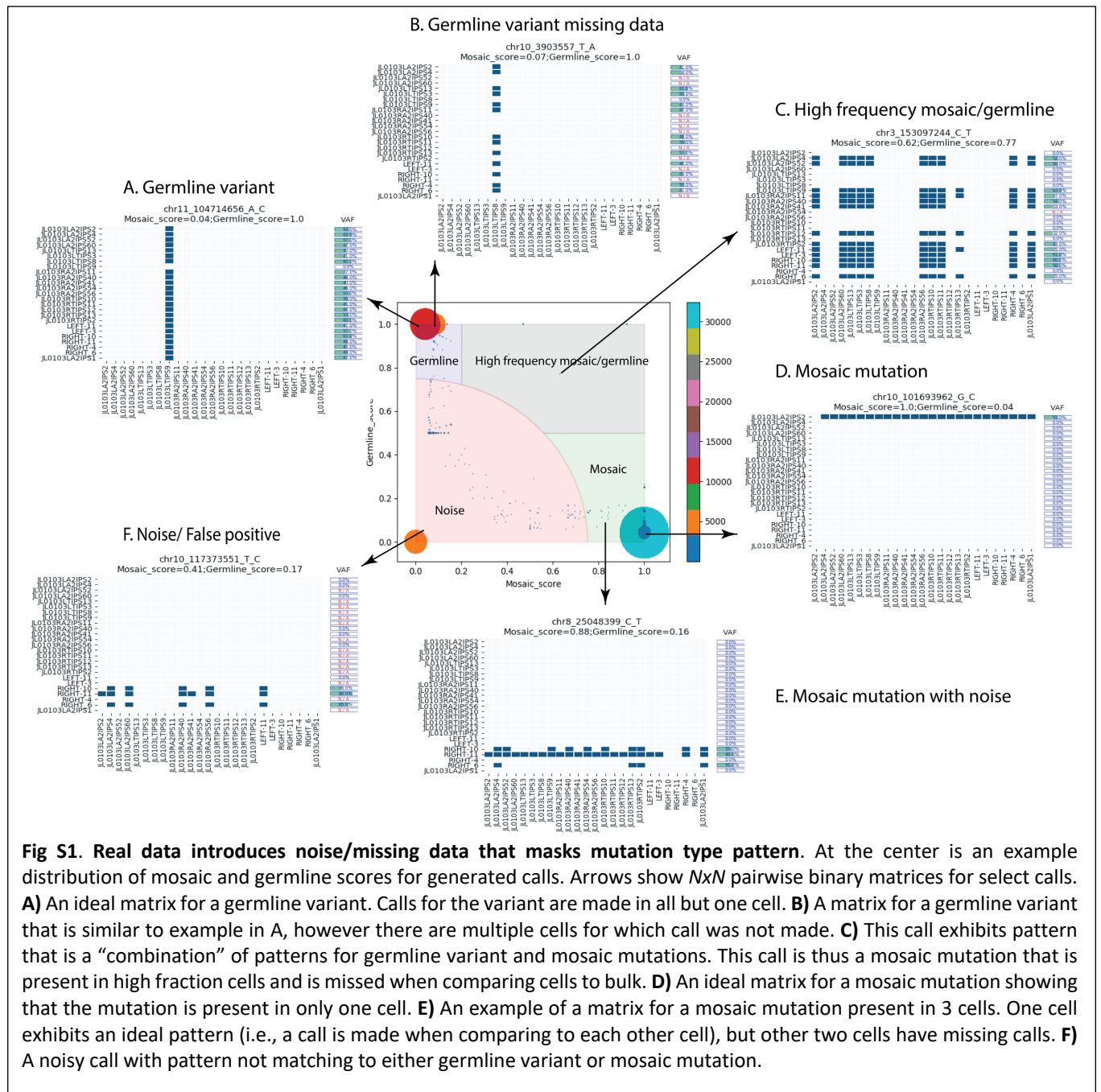

Supplement: S1 Fig — (PDF) [file pcbi.1009487.s001.pdf]

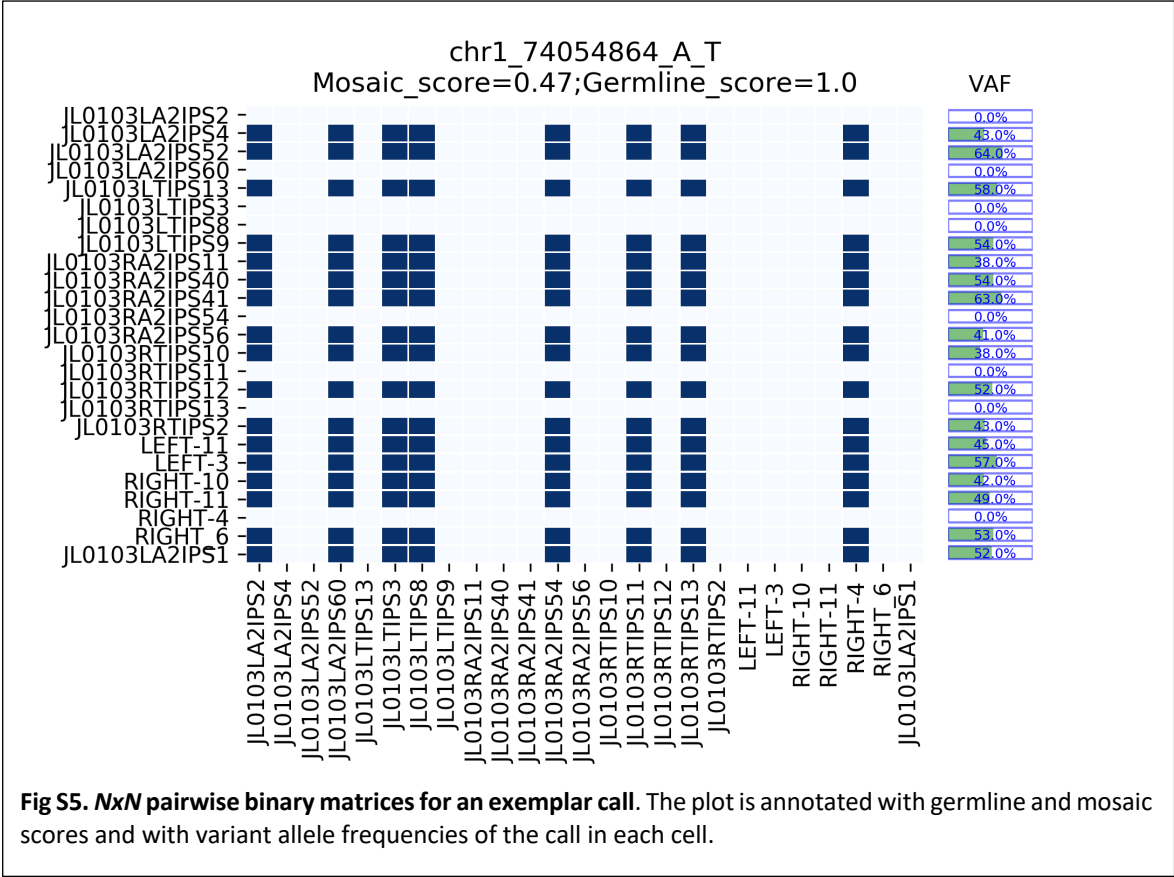

Supplement: S5 Fig — (PDF) [file pcbi.1009487.s005.pdf]

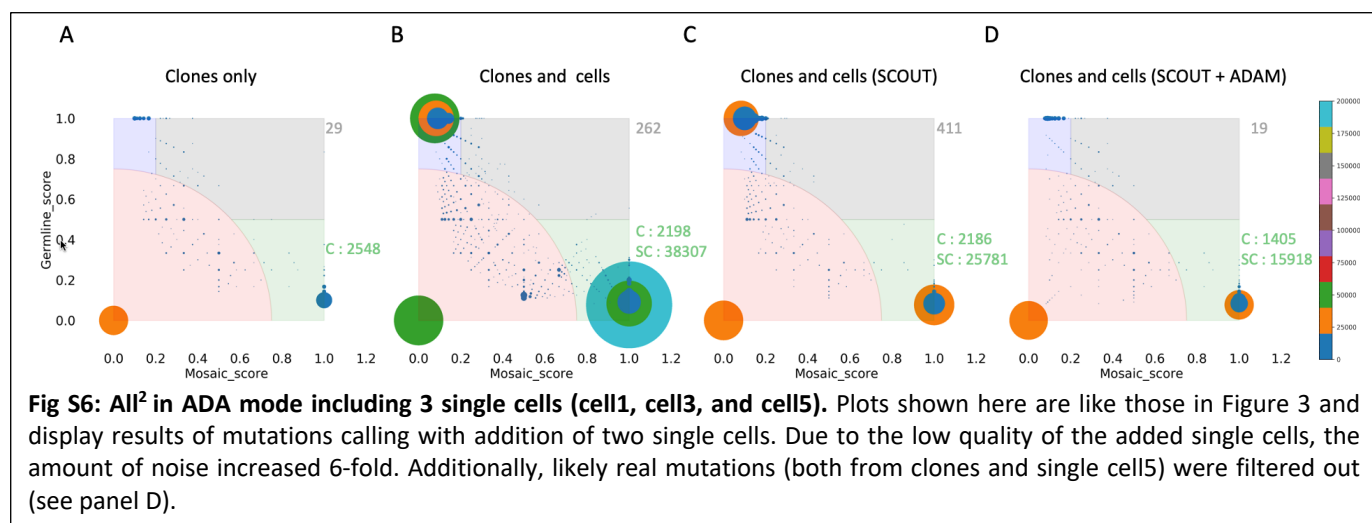

Supplement: S6 Fig — (PDF) [file pcbi.1009487.s006.pdf]
