# Supplementary material for: All2: A tool for selecting mosaic mutations from comprehensive multi-cell comparisons
Source: PLoS Comput Biol. 2022 Apr 20;18(4):e1009487. doi: 10.1371/journal.pcbi.1009487 (PMC9060341; doi:10.1371/journal.pcbi.1009487)
Supplement: S2 Fig — (PDF) [file pcbi.1009487.s002.pdf]

Number of cells (N) = 5
Number of times variant is seen (n) = 6

Mosaic mutation example

$f \approx 0.5 - \sqrt{0.25 - n/N^2} = 0.4$

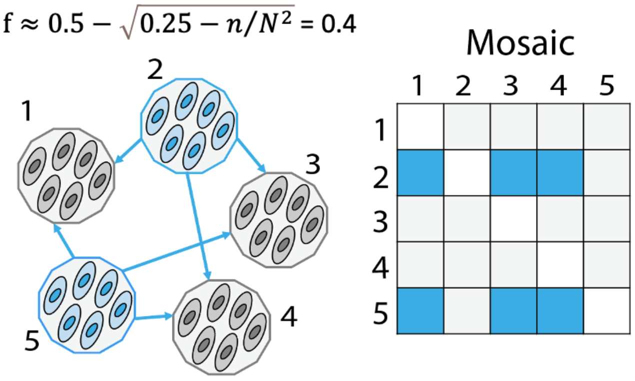

Calculation of mosaic score:

Number of cells carrying mutation (N') = f * N = 0.4 * 5 = 2
So, we take the 2 rows with maximum hits and count it

$$n_m = \sum_{i=1,2} nr_i = \sum_{i=1,5} nr_i = \sum_{i=2,3} nr_i = \sum_{i=2,4} nr_i = \sum_{i=3,4} nr_i = \sum_{i=3,5} nr_i = 3$$

$$n_m = \sum_{i=1,3} nr_i = \sum_{i=1,4} nr_i = \sum_{i=3,4} nr_i = 0$$

$$n_m = \sum_{i=2,5} nr_i = 6$$

We take the maximum possible n_m

Mosaic score = $n_m/n = \frac{6}{6} = 1$

Calculation of germline score:

Number of cells not carrying germline variant (N') = f * N = 0.4 * 5 = 2
So, we take the 2 columns with maximum hits and count it

$$n_m = \sum_{i=1,2} nc_i = \sum_{i=1,5} nc_i = \sum_{i=2,3} nc_i = \sum_{i=2,4} nc_i = \sum_{i=3,5} nc_i = \sum_{i=4,5} nc_i = 2$$

$$n_g = \sum_{i=2,5} nc_i = 0$$

$$n_g = \sum_{i=1,3} nc_i = \sum_{i=1,4} nc_i = \sum_{i=3,4} nc_i = 4$$

We take the maximum possible n_g

Germline score = $n_g/n = \frac{4}{6} = 0.6$

Fig S2. Example of calculating mosaic and germline scores for a mosaic variant.
