# Supplementary material for: All2: A tool for selecting mosaic mutations from comprehensive multi-cell comparisons
Source: PLoS Comput Biol. 2022 Apr 20;18(4):e1009487. doi: 10.1371/journal.pcbi.1009487 (PMC9060341; doi:10.1371/journal.pcbi.1009487)
Supplement: S3 Fig — (PDF) [file pcbi.1009487.s003.pdf]

Germline variant example

Number of cells (N) = 5
Number of times variant is seen (n) = 4

$f \approx 0.5 - \sqrt{0.25 - n/N^2} = 0.2$

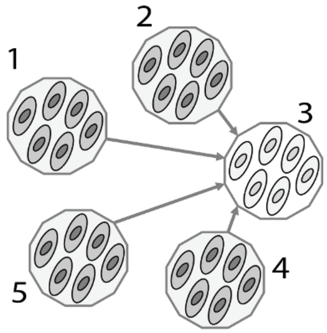

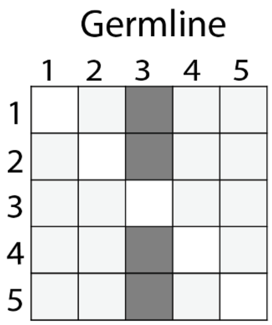

Calculation of mosaic score:

Number of cells carrying mutation (N') = f * N = 0.2 * 5 = 1

So, we take the 1 row with maximum hits and count it

$$n_m = \sum_{i=3} nr_i = 0$$

$$n_m = \sum_{i=1} nr_i = \sum_{i=2,} nr_i = \sum_{i=3} nr_i = \sum_{i=4} nr_i = \sum_{i=5} nr_i = 1$$

We take the maximum possible n_m

Mosaic score = $n_m/n = \frac{1}{4} = 0.25$

Calculation of germline score:

Number of cells not carrying germline variant (N') = f * N = 0.09 * 11 = 1

So, we take the 1 column with maximum hits and count it

$$n_g = \sum_{i=1,} nc_i = \sum_{i=2} nc_i = \sum_{i=4} nc_i = \sum_{i=5} nc_i = 0$$

$$n_g = \sum_{i=3} nc_i = 4$$

We take the maximum possible n_g

Germline score = $n_g/n = \frac{4}{4} = 1$

Fig S3. Example of calculating mosaic and germline scores for a germline variant.
