# Supplementary material for: All2: A tool for selecting mosaic mutations from comprehensive multi-cell comparisons
Source: PLoS Comput Biol. 2022 Apr 20;18(4):e1009487. doi: 10.1371/journal.pcbi.1009487 (PMC9060341; doi:10.1371/journal.pcbi.1009487)
Supplement: S4 Fig — Plots generated by All2 ‘call’ command. (PDF) [file pcbi.1009487.s004.pdf]

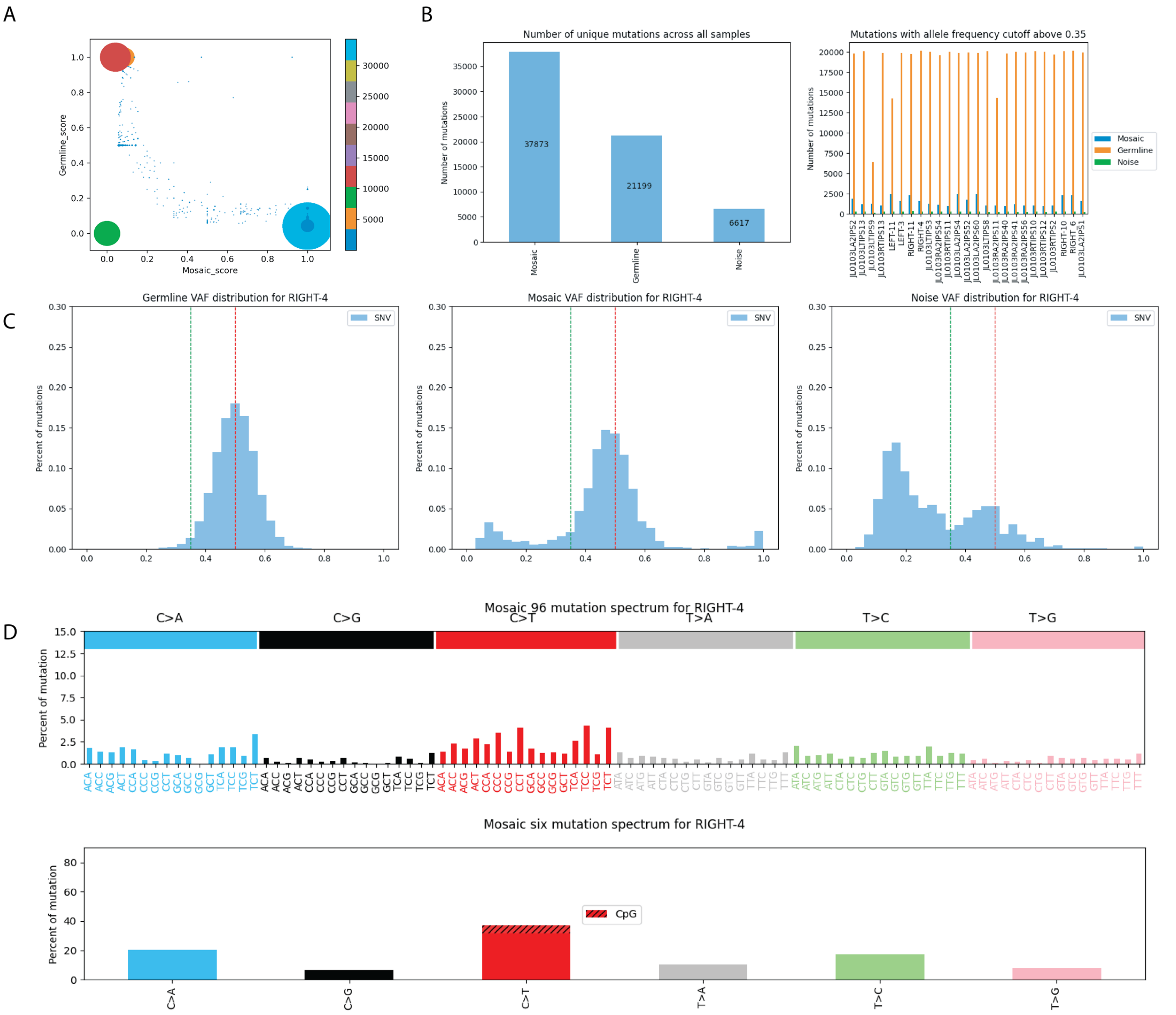

Fig S4. Plots generated by All[2] 'call' command. A) Density scatter plot showing the distribution of calls by germline and mosaic scores. This gives the user an idea on what cut-off they may want to use when running the 'call' command. **B)** Counts of call by classified type (i.e., germline variant, mosaic mutation, or false positive/noise). Calls in the summary plot (left) are redundant, e.g., the same germline variant is counted as many times as it was called in various comparisons. Call in the per cell plot (right) are by definition are non-redundant. **C)** Example of variant allele frequency (VAFs) for calls classified by types: germline variants, mosaic mutations and false positives/noise. **D)** Mutation spectra plots for mosaic mutations.
