# Supplementary material for: All2: A tool for selecting mosaic mutations from comprehensive multi-cell comparisons
Source: PLoS Comput Biol. 2022 Apr 20;18(4):e1009487. doi: 10.1371/journal.pcbi.1009487 (PMC9060341; doi:10.1371/journal.pcbi.1009487)
Supplement: S7 Fig — (PDF) [file pcbi.1009487.s007.pdf]

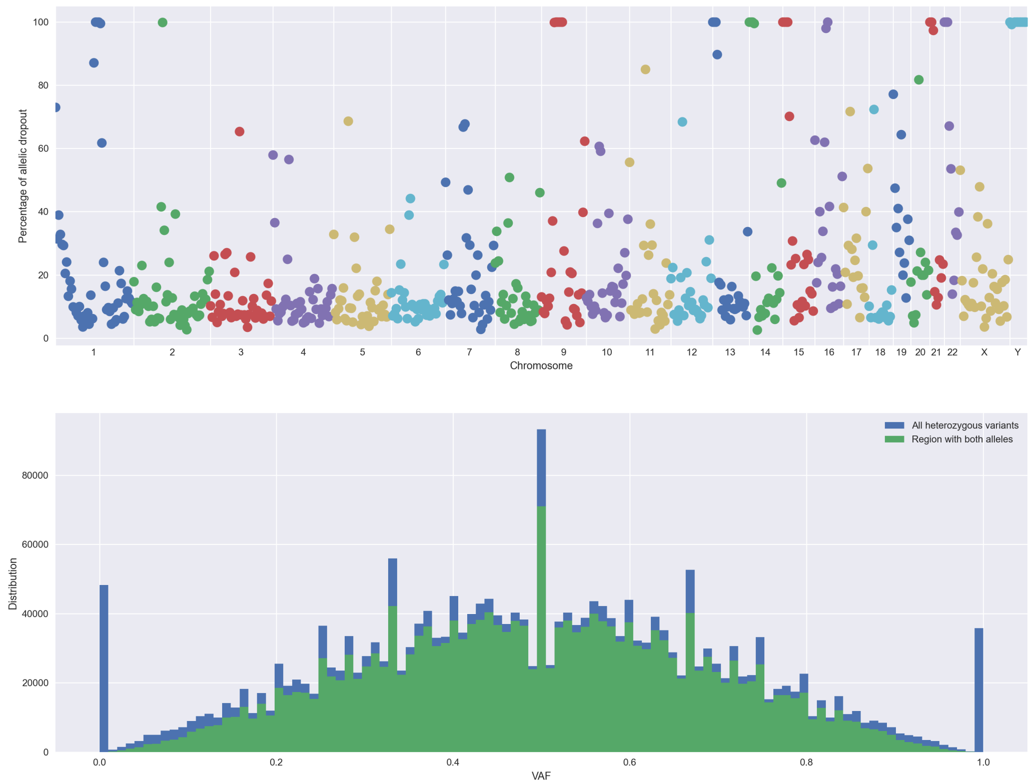

Fig S7. **Regions with allelic dropout for single cell**. Upper panel: cross genome percentage of 5 Mbp bins marked as allele drop-outs. Bottom panel: the distribution of allele frequency for heterozygous variants in all and non alle drop-outs bins.
