# Supplementary material for: All2: A tool for selecting mosaic mutations from comprehensive multi-cell comparisons
Source: PLoS Comput Biol. 2022 Apr 20;18(4):e1009487. doi: 10.1371/journal.pcbi.1009487 (PMC9060341; doi:10.1371/journal.pcbi.1009487)
Supplement: S8 Fig — (PDF) [file pcbi.1009487.s008.pdf]

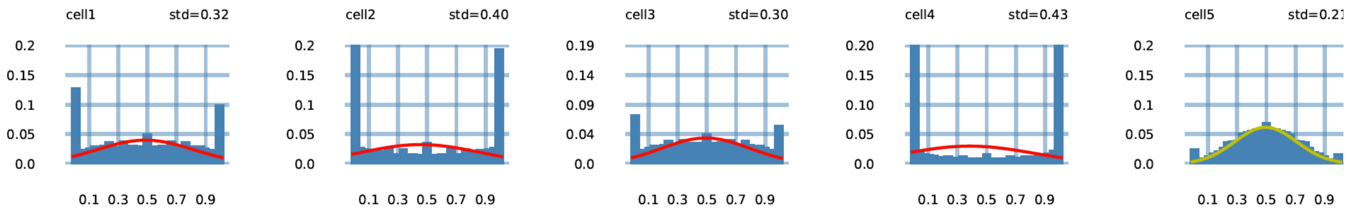

Fig S8. Allele frequency distribution of heterozygous germline variants in 5 MDA-amplified cells. Cell5 was selected for further analysis owing to its most uniform amplification and lower allele drop out as compared to the other cells.
