# Supplementary material for: All2: A tool for selecting mosaic mutations from comprehensive multi-cell comparisons
Source: PLoS Comput Biol. 2022 Apr 20;18(4):e1009487. doi: 10.1371/journal.pcbi.1009487 (PMC9060341; doi:10.1371/journal.pcbi.1009487)
Supplement: S1 Table — (PDF) [file pcbi.1009487.s009.pdf]

Samples	Number of SNV in tree	SNVs with coverage > 200X	# of SNVs with VAF >= 4% < 35%	# of SNVs with VAF <= 1%	Missed	Missed(As fraction)
LA1	65	50	14	24	12	24.00%
LA2	65	49	10	15	24	48.98%
RA1	65	51	0	19	32	62.75%
RA2	65	51	10	15	26	50.98%
LT	65	51	22	12	17	33.33%
RT	65	50	14	21	15	30.00%
Blood	65	50	16	19	15	30.00%
Saliva	65	50	17	12	21	42.00%

Table S1. Fraction of mosaic mutations (SNVs) missed using different tissue types. From capture sequencing of 65 SNVs from the lineage tree (Figure 2B), we only considered the one with coverage of 200X or higher. SNVs with VAF greater than 4% and less than 35% represents mutations that can be derived from bulk where as SNVs which VAF less than 1% can be derived from bulk to single cell paired comparison. By subtracting the number of SNVs that can be found by bulk analyses and paired analyses from the number of "SNVs with coverage > 200X" we can calculate the fraction of missed SNVs. The column "Missed (As Fraction)" shows that LA1, LT and RT have comparable missed SNVs with blood and saliva, whereas in LA2, RA1 and RA2, half of the SNVs are being missed. These results suggest that using any of the bulk samples does not outperform ALL^2 for lineage tree construction.
